# Sintilimab combined with axitinib in the treatment of advanced chromophobe renal cell carcinoma: a case report

**DOI:** 10.3389/fonc.2024.1325999

**Published:** 2024-02-02

**Authors:** Huimin Zhang, Xiaofeng Cong, Chen Chen, Ziling Liu

**Affiliations:** Cancer Center, The First Hospital of Jilin University, Changchun, China

**Keywords:** chromophobe renal cell carcinoma, sintilimab, axitinib, second-line treatment, long survival

## Abstract

Chromophobe renal cell carcinoma (ChRCC) is a rare pathological type of renal cell carcinoma (RCC). Related systematic studies involving large numbers of patients are lacking, and more importantly, there is currently no international consensus on post-line treatment guidelines for ChRCC. The rapid development of systemic treatment with molecular targeted therapies and immune checkpoint inhibitors has brought effective approaches for patients with clear cell renal cell carcinoma (ccRCC), while progress in the treatment of ChRCC is still limited. In this case report, the patient was initially diagnosed at the early stage; 4 years post-surgery, she developed lung metastases and the disease progressed once again after being treated with sunitinib monotherapy for 3 years. However, after combining the immunotherapy sintilimab with the targeted therapy axitinib as second-line treatment, imageological examination showed lesions in the lungs that gradually decreased, and the bone metastases remained stable. To date, the patient has been continuously treated for over 2 years and is still undergoing regular treatment and follow-up. This case is the first to report the long-term survival of metastatic disease by using this treatment regimen and to propose a potential therapeutic option for patients with metastatic ChRCC. Since only one case was observed in this report, further study is needed.

## 1 Introduction

Renal cell carcinoma, ranking third in morbidity of urinary system tumors, accounts for 2%–3% of all malignant tumors (1). Chromophobe renal cell carcinoma is the third most common pathological type of RCC following clear renal cell carcinoma and papillary renal cell carcinoma, representing approximately 5%–7% of RCC (2). Distinct from clear cell renal cell carcinoma (ccRCC) which originates from the proximal convoluted tubule, chromophobe renal cell carcinoma (ChRCC) is derived from the cells of the distal convoluted tubules and collecting tubules of the nephron. In terms of biological behavior, ChRCC presents the characteristic of low-malignant potential tumors with a 5%–6% risk of metastasis, showing a better prognosis than those of other subtypes of RCC (2), whereas the prognosis of metastatic ChRCC is worse than that of advanced ccRCC (3). In the context of RCC, most advances have been made to enhance the therapeutic efficacy of ccRCC. Moreover, due to the rarity of ChRCC, there are still very few clinical trials and reports with large sample sizes. Therefore, the therapeutic regimen of ChRCC is normally extrapolated from ccRCC studies (4). For ChRCC diagnosed at stages I–III, surgery is considered the gold-standard treatment (5). Moreover, adjuvant therapy has no additional benefit for these patients (6). Nevertheless, for advanced or recurrent ChRCC, there is limited clinical evidence to guide the optimal choice of treatments, and the approval of systemic therapies such as tyrosine kinase inhibitors, anti-angiogenic inhibitors, and immune checkpoint inhibitors has been applied as palliative treatment for those patients. In this case report, we will present a long-term survival case with metastatic ChRCC treated with sintilimab in combination with axitinib as a second-line treatment, which serves as a valuable therapeutic reference for doctors to manage ChRCC patients.

## 2 Case presentation

**Figure 1:**
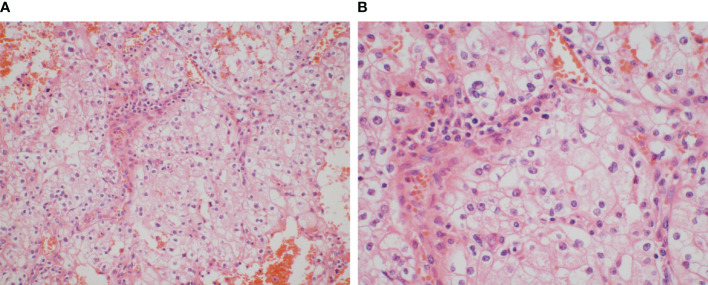
Images were taken using objectives **(A)** at ×20 magnification and **(B)** at ×40 magnification. Tumor cells show obvious atypia, arranged in a sheet-like distribution with nests, and the abundant blood vessels are between the tumor nests. The tumor cells appear to have a clear cytoplasm with weak cytoplasmic staining and clear cell borders, showing a plant cell-like appearance, and they also contain an eosinophilic cytoplasm and irregular nuclear contours with a perinuclear halo, and rough chromatin and obvious nucleoli can be observed.

A 59-year-old woman with no significant past medical history was admitted in February 2014 since abdominal ultrasonography performed at the routine health check-up indicated a “space-occupying lesion in the right kidney.” The patient was afebrile and had no abdominal or lower back pain. She also had no symptoms such as frequent urination, painful urination, urgency visible hematuria, or weight loss. No positive signs were identified on the gross examination of the abdominal cavity. Subsequent computed tomography (CT) showed a mass-like abnormally enhanced shadow approximately 5.4 × 4.6 cm in size protruding outward that was seen in the lower pole of the right kidney. Enhanced CT examination in the cortical phase showed uneven enhancement and uneven hypoenhancement in the renal parenchymal enhancement phase, and the boundary between the lesion and the local renal calyces was unclear. Consequently, laparoscopic radical right nephrectomy was performed in March 2014. The postoperative pathological report showed the following: tumor cells showing obvious atypia, which are arranged in a sheet-like distribution with nests; abundant blood vessels between the tumor nests; tumor cells that appear to have a clear cytoplasm with weak cytoplasmic staining and clear cell borders, showing a plant cell-like appearance; tumor cells containing an eosinophilic cytoplasm and irregular nuclear contours with perinuclear halo; and rough chromatin and obvious nucleoli (**Figure 1**). The lesion in the right kidney was diagnosed as ChRCC with Fuhrman nuclear grading III. The tumor was confined to the renal capsule without invading the renal pelvis, the perinephric fat, or the renal blood vessels. Therefore, she was demonstrably diagnosed at T1bN0M0. The patient received no antitumor treatment after the surgery; instead, she performed regular postoperative instrumental checks.

**Figure 2:**
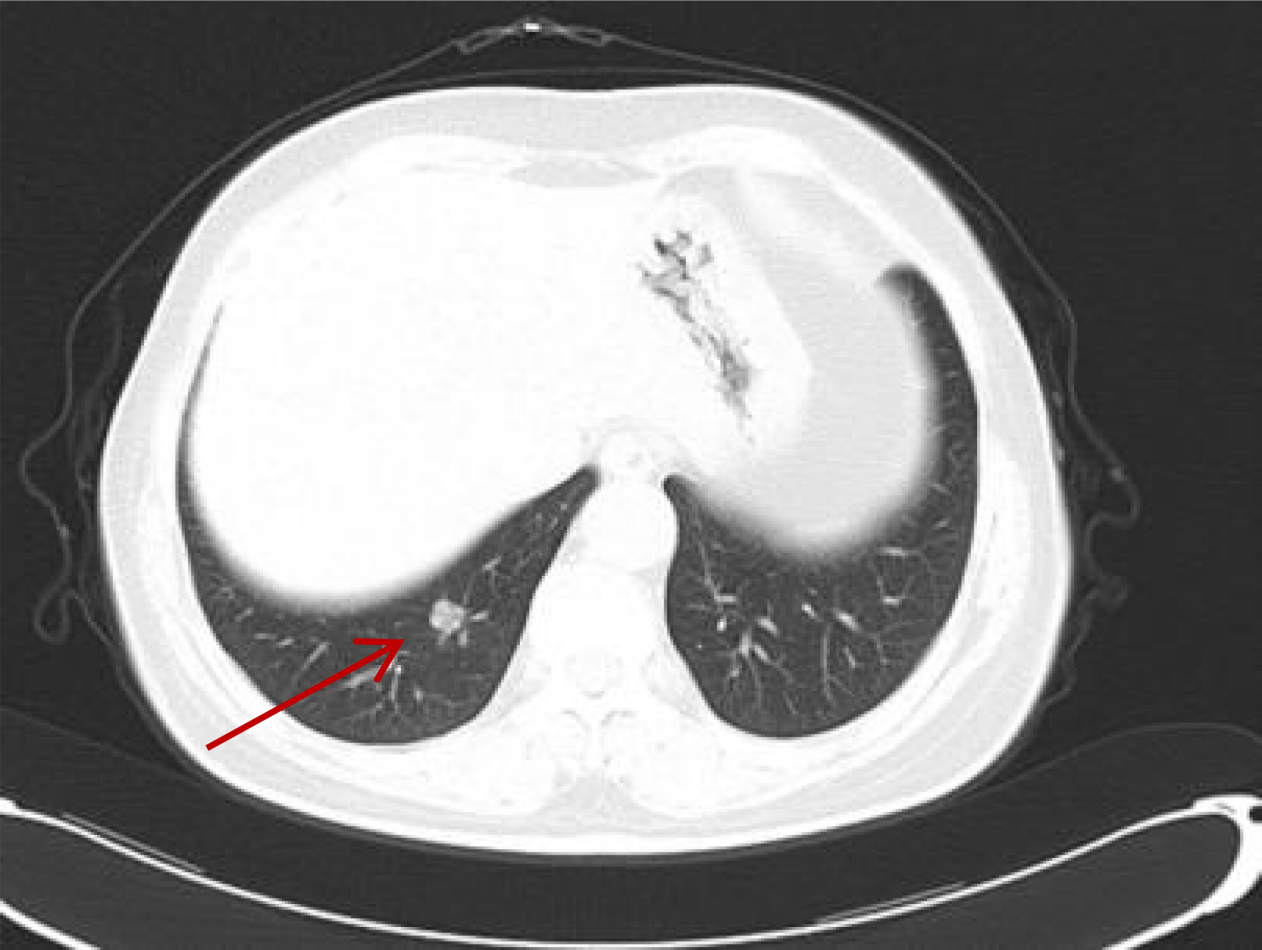
On 23 March 2018, CT showed metastatic lesions in the lungs 4 years post-surgery. Multiple nodular high-density shadows were observed in the lungs, and the largest one was approximately 0.2–0.9 cm in size.

On 23 March 2018, a CT scan of the lungs demonstrated that multiple nodular high-density shadows approximately 0.2–0.9 cm in size were observed in the lungs, which were considered as metastatic malignant tumors (**Figure 2**). Moreover, no malignancy was identified on the abdomen CT. Thus, she received sunitinib at a dose of 37.5 mg orally for 4 weeks on and 2 weeks off-cycle as first-line treatment since 25 March 2018, and efficacy was assessed as stable disease (SD) during regular treatment according to RECIST tumor evaluation criteria.

**Figure 3:**
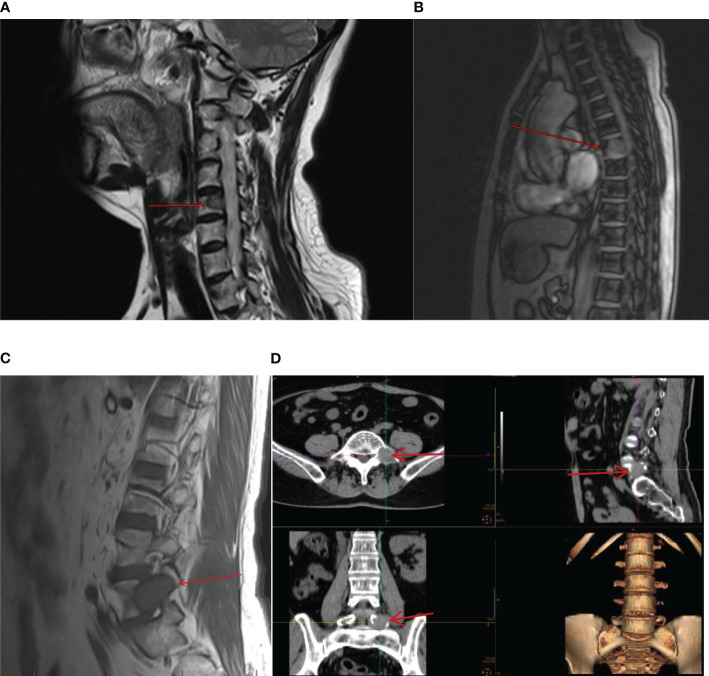
On 22 February 2021, CT and MRI showed new metastatic lesions in the bone after being treated with sunitinib monotherapy for 3 years in the cervical vertebra **(A)**, thoracic vertebra **(B)**, lumbar vertebra **(C)**, and space-occupying lesion next to the left side of L5 **(D)**.

**Figure 4:**
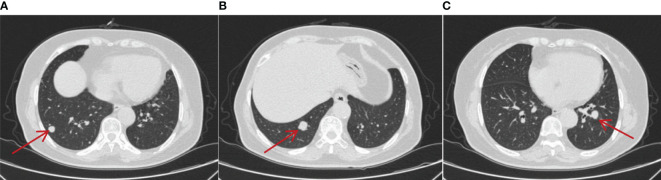
On 22 February 2021, CT showed the metastatic lesions enlarged in different lung lobes **(A–C)** after being treated with sunitinib monotherapy for 3 years. Multiple nodular highdensity shadows were observed in both lungs, and the largest was approximately 0.4–1.5 cm in size.

However, on 22 February 2021, the patient was admitted to the spinal surgery department because she presented with waist and buttock pain and lower limb numbness with pain without obvious triggers, which would worsen after exertion and resolve after rest. The performance of neurological examination suggested that she had limitations in waist mobility and, spontaneously, an increase in pain on percussion from the fourth lumbar vertebra to the sacral vertebra. Furthermore, the sensation of the posterolateral surface of the calf decreased, and a positive straight leg raise test of the left limb was recorded. Magnetic resonance imaging (MRI) test of the cervical, thoracic, and lumbar vertebrae and CT of the lumbar and sacral vertebrae indicated abnormal signals in C5, T1, T6–10, and T12, which should be speculated as bone metastasis. Furthermore, there were space-occupying lesions next to the left side of L5 and in the right alae sacralis, which were suspected as metastatic tumors (**Figure 3**). Furthermore, pulmonary CT showed multiple nodular high-density shadows in both lungs approximately 0.4–1.5 cm in size, and multiple lymph nodes were enlarged in the mediastinum and bilateral pulmonary hilum with the largest one being 1.5 cm in size (**Figure 4**). In addition, low-density shadows presented on the right 4th and 6th ribs. All these abnormal signals were considered as metastatic tumors and the patient’s condition progressed significantly.

**Figure 5:**
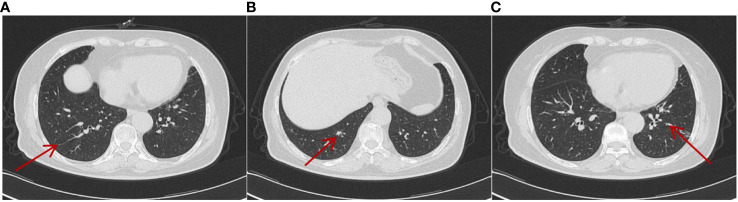
On 25 October 2023, CT showed that metastatic lesions decreased in size in different lung lobes **(A–C)** (by comparing with corresponding parts in **Figure 4**) after being treated with sintilimab plus axitinib for over 2 years, and the largest one was approximately 0.2–0.7cm in size. No obvious enlargement of the lymph nodes in the mediastinum was observed.

**Figure 6:**
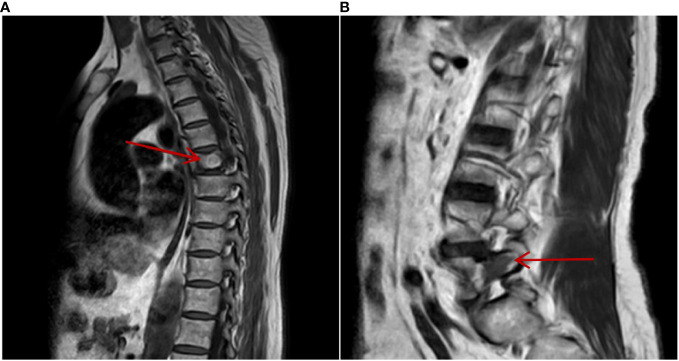
On 3 March 2023, MRI of the thoracic and lumbar vertebrae showed that metastatic lesions in the thoracic vertebra **(A)** and lumbar vertebra **(B)** bone remained stable after being treated with sintilimab plus axitinib for 2 years.

Aiming to relieve symptoms of pain, a “spinal nerve root block” was performed on 25 February 2021. In terms of antitumor comprehensive treatment, the patient was administered two doses of intravenous sintilimab (200 mg) every 3 weeks, a dosage of 10 mg/day of axitinib divided into two doses orally, and simultaneously denosumab by subcutaneous injection (120 mg) every 3 weeks as second-line therapy from 26 February 2021. Axitinib-related toxicities included grade 3 hypertension and grade 2 fatigue and body aches, which were treated appropriately by symptomatic treatment. Hypertension was treated with nifedipine controlled-release tablets 30 mg qd and sacubitril/valsartan sodium tablets 50 mg bid, and no axitinib dosage was changed due to adverse reactions. Furthermore, fatigue was treated with coenzyme Q10 and supplements, and non-steroidal anti-inflammatory drugs were occasionally used for pain relief. During the treatment, the tumors in the lungs gradually shrank, and the most recent pulmonary CT was conducted on 25 October 2023, which showed multiple nodular high-density shadows in the lungs with the largest one approximately 0.2–0.7 cm in size. Meanwhile, no obvious enlargement of the lymph nodes in the mediastinum was observed, and bone metastases on the right 4th and 6th ribs were similar to the former ones (**Figure 5**). Moreover, on 3 March 2023, MRI of the thoracic and lumbar vertebrae revealed that abnormal signals were roughly similar to the previous image (**Figure 6**). Accordingly, the overall evaluation showed partial response (PR) during regular treatment according to RECIST. Up to December 2023, the patient has accepted 43 cycles of immunotherapy combined with targeted therapy and is still receiving regular treatment and follow-up.

## 3 Discussion

ChRCC was first reported by Thoenes in 1985 whose designation came from the observation of peculiar histomorphology. In general, ChRCC is mainly categorized into the following major subtypes: 1) classical, 2) eosinophilic, 3) sarcomatoid, and 4) other rare patterns (7). The unique genetic characteristic of ChRCC is the loss of entire copies of chromosomes including 1, 2, 6, 10, 13, and 17, which contributes to improving diagnostic accuracy (8). Furthermore, the somatic mutation rate of ChRCC is very low except for TP53 and PTEN, which are the two most common mutation genes (9), especially in patients with metastatic or sarcomatoid ChRCC (2, 10). The combination of next-generation sequencing (NGS) analysis, gene expression profile, high-throughput SNP genotyping, and pathway analysis has confirmed that signal pathways of c-erbB2 (HER2) and mammalian target of rapamycin (mTOR) signaling are dysregulated in ChRCC (11, 12), which provides a biological basis for targeted therapy.

In terms of clinical features, unlike ccRCC, patients who develop ChRCC present at a younger age, and it usually affects female patients (13). Remarkably, the characteristic triad of low back pain, gross hematuria, and palpable abdominal mass rarely occurs in ChRCC, and most patients are asymptomatic at the time of diagnosis. Therefore, the majority of renal masses are incidentally discovered by imaging modalities (4), which is consistent with our case. Patients with stage I–III tumors generally have a good prognosis with approximately 89.3% of 5-year recurrence-free survival rate and 93% of 5-year cancer-specific survival (14), and 6%–7% of ChRCC patients show advanced disease, which mainly affects the liver or lungs (15). In the treatment of advanced or recurrent ChRCC, due to the relative rarity of this malignancy, the pathogenesis is, however, not well described especially in the advanced stage. Although ChRCC is almost completely different from ccRCC regarding its response to targeted therapies and immune checkpoint inhibitors (16), the favorable responses of tyrosine kinase inhibitors and mTOR inhibitors have been reported in clinical trials such as ESPN and ASPEN (17, 18), which provide a reference value for ChRCC patients although the number of patients included in these trials was limited, and everolimus and sunitinib were both considered the drugs of choice for ChRCC patients. Therefore, sunitinib was chosen as the first-line treatment for this patient and lesions were well controlled during the treatment.

It is a pity that the pulmonary lesions were enlarged than before, and simultaneously, multiple bone metastases occurred after the oral administration of targeted drugs for almost 3 years, which was considered resistance to targeted drugs. After the surgery that relieved the symptoms, we faced complex choices when making decisions about the second-line drugs for antitumor treatment, since there is currently no consensus in the latest guidelines regarding the optimal treatment. The introduction of immunotherapy has drastically improved the prognosis of patients with advanced ccRCC, which contributes to the transformation of the treatment mode of metastatic ccRCC, and immunotherapy combined with targeted therapy is one actionable option after targeted therapy failure. However, immune checkpoint inhibitors have uncertain antitumor activity in ChRCC, and prospective studies evaluating the safety and efficacy of immune checkpoint blockade are still ongoing. The KEYNOTE-427 study explored the antitumor activity of pembrolizumab in the treatment of patients with non-clear cell renal carcinoma. Twenty-one (13%) of the 165 patients enrolled had ChRCC, and 50% of the total patients responded within 12 months. Moreover, the objective response rate (ORR) was 9.5% in the ChRCC subgroup (19), which hints at the limited efficacy of immune checkpoint blockade in the treatment of ChRCC. As a matter of fact, PD-L1 expression was only detected in 35% of the cases in the entire cohort, which might affect the response rate of immunotherapy. In addition, a phase II trial assessed the effectiveness of cabozantinib plus nivolumab in patients with non-clear cell renal carcinoma (NCT03635892). Seven ChRCC patients were enrolled in cohort 2, none of whom achieved an objective response. The disease control rate was 71% [95% confidence interval (CI): 29.0–96.3]. Moreover, the clinical benefit rate was 29% (95% CI: 3.7–71.0) (20), which also obtained unsatisfactory results. It is worth noting that sarcomatoid differentiation in RCC has been verified to be an indicator of limited therapeutic response and worse prognosis (21). Despite this, PD-L1 expression and the level of tumor-infiltrating lymphocytes in sarcomatoid RCC appear to be higher compared with standard RCC without sarcomatoid differentiation, exhibiting a unique immunological molecular background and providing new clinical opportunities (22). However, in our patient, light microscopic study showed no evidence of sarcomatoid histology. Combined with our previous experience in ccRCC, we chose to combine immunotherapy with targeted therapy as the second-line drug for patients. In terms of immunotherapy, factors such as medicine availability as well as cost-effectiveness performance were taken into consideration. Here, we explained in detail to the patient the current approval for advanced RCC such as pembrolizumab or nivolumab, and she was willing to use immunotherapy but simultaneously hesitated because of the financial burden; thus, she consulted repeatedly about cost-effective medications. After reviewing relevant literature, sintilimab combined with axitinib achieved a preferable objective response rate for advanced RCC with intermediate/high risk in a single-center study, which included three non-ccRCC patients. For the whole cohort, the disease control rate was 90% (9/10), and the objective response rate was 40% (4/10) (23). Sintilimab was a fully recombinant human IgG4 anti-PD-1 monoclonal antibody, which can restore the endogenous antitumor T-cell response by binding to PD-1, therefore blocking the binding and interaction between PD-1 and its ligands (PD-L1 and PD-L2). It has significantly improved patient survival outcome and has shown a lower incidence of treatment‐related adverse events and superior tolerability in multiple types of malignancies, but insufficient data are available on RCC. We iteratively emphasized to the patient the rarity of the pathological type and the uncertainty about the treatment efficacy. She insisted on receiving this affordable immunotherapy; therefore, sintilimab was selected after fully communicating with the patient. In recent years, there has been a single-center study that verified its effectiveness as a second-line treatment for advanced ccRCC, which strengthened the interpretability of our use (24). As for targeted therapy, since lung metastases progressed, which was treated by sunitinib for almost 3 years, we thought she developed resistance to sunitinib and this drug could not effectively continue to control the lesions; therefore, sunitinib needed to be switched to another drug. Based on our experience managing advanced RCC, axitinib, a highly selective VEGFR1, 2, and 3 inhibitor, characterized by high target affinity, strong specificity, and mild adverse reactions compared with other tyrosine kinase inhibitors, was determined as the second-line oral treatment. In the meantime, denosumab injection, a human monoclonal antibody targeting the bone resorption mediator RANKL, was regularly given to treat bone metastasis. During treatment, the patient was regularly scheduled with clinical and radiographic examinations, and the lung lesions gradually became smaller than before and bone lesions remained stable, which were evaluated as PR. By the end of December 2023, the patient has completed 43 courses of immunotherapy combined with targeted therapy and is still receiving regular treatment and follow-up, achieving long survival with cancer. Although the antitumor treatment is impressively effective, there are toxicity and side effects of axitinib that troubled the patient during medication such as grade 3 hypertension, grade 2 fatigue, and body aches that were managed accordingly, which should be taken seriously in clinical treatment, since it can be challenging due to instances of side effects from the oral drug. The imaging review outcomes confirmed the patient’s determination for this treatment, and she is very satisfied with the current treatment plan in terms of its less burden on financial performance; therefore, her compliance is high, treatment is regular, and the patient shows no signs of rejection due to the side effects of medication.

At present, there is still limited knowledge about the surveillance and optimal management of patients with recurrent ChRCC. In our case, sintilimab combined with axitinib achieved significant efficacy, which suggests that this therapeutic schedule may be a potential effective treatment option for patients with metastatic ChRCC and can provide some data reference for doctors. What is noteworthy is that the side effects during the oral administration of axitinib remind us that sufficient attention should be paid to drug-related adverse reactions and proactive decisions should be made actively to handle them since these cumulative side effects may lead to treatment interruption that affects effective antitumor treatment; therefore, proper management of these adverse events will ensure optimal survival benefits for patients. In addition, based on the unique immunological background of some ChRCC with sarcomatoid differentiation, the application of immunotherapy may provide advantageous therapeutic opportunities for comprehensive treatment. Given the rarity of ChRCC, therefore, we actively recommend its inclusion in clinical trials since it seems that ChRCC is likely a separate entity with unique disease mechanisms, which may need to be treated with different therapeutic strategies to improve efficacy, and future studies will provide more insights regarding the precise molecular mechanisms. Since only one case of sintilimab combined with axitinib in the treatment of advanced ChRCC has been observed in this report, the clinical data are very limited. Further follow-up and accumulation of more experience are required, and the efficacy and safety of this regimen need further verification. Moreover, we have to acknowledge the limitations of this case concerning more consummate genetic information and more detailed immunohistochemistry information since the patient was diagnosed a long time ago and there were technical limitations at that time.

## Data availability statement

The original contributions presented in the study are included in the article/supplementary material. Further inquiries can be directed to the corresponding author.

## Ethics statement

The studies involving humans were approved by The Ethics committee of the First Hospital of Jilin University. The studies were conducted in accordance with the local legislation and institutional requirements. The participants provided their written informed consent to participate in this study. Written informed consent was obtained from the individual(s) for the publication of any potentially identifiable images or data included in this article.

## Author contributions

HZ: Writing – original draft. XC: Writing – original draft. CC: Writing – review & editing. ZL: Writing – review & editing, Project administration.
